# Observations on the Influenza or Epidemic Catarrh, as It Appeared at Bristol and Its Environs, during the Months of May and June 1782. To Which Is Added a Meteorological Journal of the Weather

**Published:** 1782

**Authors:** 


IX. *Obſervations on the Influenza or epidemic Catarrh, as it appeared at Briſtol and its environs, during the Months of May and June* 1782. *To which is added a meteorological Journal of the Weather. By* A. Broughton, M. D. *Fellow of the Royal Medical Society of Edinburgh, and one of the Phyſicians to the Briſtol Infirmary.* 8vo. Robinſon, *London.* 31 pages. 1s.

FROM the beſt information which this writer was able to obtain, the epidemic made its appearance at Briſtol about the ſecond week in May; and after raging with great rapidity during the remainder of that month, about the middle of June it began to abate, and by the end of it, or the beginning of July, it ſeemed to have entirely diſappeared.

Some have ſuppoſed that the diſeaſe was owing to the backwardneſs of the ſpring, in conſequence of which the air contained a ſuperabundant quantity of phlogiſton; but from ſome experiments made at Briſtol by our author's friend, Mr. Becket, it appears that the air, at the time when the diſeaſe raged moſt, contained no more phlogiſton than what is uſual. Dr. Broughton thinks it not at all improbable, but that the large quantity of rain which fell ſome weeks previous to its appearance might cauſe ſuch a great degree of evaporation from the ſurface of the earth as to produce a cold ſufficient to conſtringe the pores on the ſurface of the body, and that the great conſent between the exhalation from the lungs and the cutaneous perſpiration may account for its particular determination to thoſe parts. This argument is ingenious, but in the preſent inſtance we cannot adopt it. There were phenomena in the late epidemic, which we think can be explained in no other way than by ſuppoſing it to have been a contagious diſeaſe.

Dr. Broughton gives a very exact hiſtory of the ſymptoms of this epidemic. Theſe ſymptoms were in general ſimilar to thoſe deſcribed in the preceding article. In ſome patients, we are told, the diſorder was attended with pains in different parts of the body, reſembling the rheumatiſm. In others the tonſils appeared enlarged and ſlightly inflamed; but in none of thoſe, who came within our author's obſervation, did the irritation ſeem to extend itſelf beyond the trachea. Two patients, on the ſecond day of the diſeaſe, complained of a difficulty in paſſing water, which gradually increaſed as the ſymptoms toms advanced, and went off on the body's returning to its uſual ſtate.

Bleeding ſeemed to do harm, at leaſt our author ſuſpects that all thoſe, treated in this manner, were much longer in recovering than thoſe in whom the lancet was not uſed. Where the inflammatory ſymptoms, as ſometimes happened, were conſiderable, a purgative remedy, ſuch as the *decoct. tamar. cum ſena*, generally procured ſome remiſſion. Where the ſymptoms of fever ran very high, with great oppreſſion and difficulty of breathing, a ſolution of emetic tartar, ſo as to produce vomiting, ſeemed to afford much relief, and where it excited ſome degree of diaphoreſis the good effect was ſtill more evident. It was not, however, in every caſe that either of theſe remedies could be adminiſtered; for ſome on the firſt attack complained of pain in the bowels attended with a diarrhœa, and ſuch were only to be relieved by opiates.

After the belly had been gently opened by a purgative remedy, the uſe of mild diaphoretics ſeemed to afford much relief. To the uſe of nitre in caſes attended with a cough our author has an objection on account of its ſtimulating property.

On the going off of the diſeaſe it often happened, that the matter ſecreted by the glands about the bronchiæ was ſo thick that it was with difficulty the patient could ſpit it up; in ſuch caſes, we are told, the *lac fœtidum*, or *lac ammon.* afforded relief; and if theſe failed bliſters were applied between the ſhoulders.

The work cloſes with an extract from a meteorological journal, from the 20th of April to the 20th of June 1782, kept at Briſtol by Mr. Becket.

